# AI-driven spatial analysis of tumor-infiltrating lymphocytes predicts chemo-immunotherapy response in triple-negative breast cancer

**DOI:** 10.3389/fimmu.2026.1797753

**Published:** 2026-04-16

**Authors:** Hai Liang, Hejie Zhu, Liwei Wang, James Yu, Ru Feng, Xiaoshuang Yan, Liang Shi, Jiayi Xiao, Huirong Zhou, Peilin Chen, Hao Liu, Linfeng Zheng

**Affiliations:** 1 Shanghai Qilu Pharmaceutical R&D Center Ltd., Shanghai, China; 2 Product Development Department, SODA Data Technology Inc., Shanghai, China; 3 Department of Pathology, Zhejiang Cancer Hospital, Hangzhou Institute of Medicine (HIM), Chinese Academy of Sciences, Hangzhou, China

**Keywords:** artificial intelligence, Miller-Payne, tertiary lymphoid structures, triple-negative breast cancer, tumor-infiltrating lymphocytes

## Abstract

**Introduction:**

Triple-negative breast cancer (TNBC) is an aggressive subtype with limited therapeutic options due to lack of estrogen receptor (ER), progesterone receptor (PR), and human epidermal growth factor receptor 2 (HER2) expression. While neoadjuvant immunotherapy shows promise, the impact of spatial organization of tertiary lymphoid structures (TLS) and tumor-infiltrating lymphocytes (TILs) on treatment efficacy remains incompletely characterized. This exploratory study aims to establish an AI-based quantitative framework for analyzing tumor-immune spatial interactions and develop a predictive model for chemo-immunotherapy response.

**Methods:**

We developed an integrated AI pipeline for automated analysis of hematoxylin-eosin (HE)-stained breast cancer samples. The framework incorporates: 1) TLS detection and classification, 2) TIL quantification, and 3) spatial relationship mapping between lymphocytes and tumor cells. Biomarkers associated with Miller-Payne (MP) response grades were identified using multivariate statistics, enabling construction of a random forest prognostic model.

**Results:**

The TLS recognition model demonstrated substantial agreement with pathologists (κ=0.73), while the TIL classifier achieved 0.92 accuracy. Analysis of 32 HE images from triple-negative breast cancer (TNBC) cases treated with neoadjuvant chemo-immunotherapy revealed significant associations: stromal lymphocyte density and percentage positively correlated with MP grades (p<0.05); average cell counts in 2-cell and 3-cell lymphocyte aggregates showed no significant correlations; shorter mean minimum distances between lymphocytes and tumor cells within 10-30 μm radius range were inversely associated with MP grades (p<0.05). The spatial-feature-based prediction model achieved an AUC of 0.81 (95% CI: 0.76–0.86).

**Conclusions:**

This study established an AI-driven HE analysis pipeline that precisely quantified spatial determinants of tumor-immune interactions. Lymphocyte spatial organization, particularly proximity to tumor cells and lymphocyte aggregation patterns, serves as a critical predictor of chemo-immunotherapy response beyond density metrics. The validated MP-grade prediction model demonstrates translational potential for clinical decision-making in TNBC management, although external validation in larger cohorts is warranted.

## 1 Introduction

Triple-negative breast cancer (TNBC) represents a highly aggressive breast cancer subtype characterized by poor prognosis. Due to the absence of estrogen receptor (ER), progesterone receptor (PR), and human epidermal growth factor receptor 2 (HER2) expression, this subtype exhibits limited responsiveness to conventional endocrine and targeted therapies. Chemotherapy remains the primary treatment for triple-negative breast cancer (TNBC); however, its efficacy is limited by suboptimal outcomes, including persistently high recurrence and mortality rates (1). Consequently, identifying novel therapeutic targets and biomarkers is critical for improving clinical outcomes in TNBC patients.

Recent advances in immunotherapy have yielded new treatment options. Immune checkpoint inhibitors (ICIs), particularly through combination regimens and novel therapeutic strategies, have achieved significant clinical advances. The phase III KEYNOTE-522 trial revealed that pembrolizumab (a PD-1 inhibitor) combined with chemotherapy significantly enhanced pathological complete response (pCR: 64.8% vs. 51.2%) and event-free survival (EFS) in early-stage TNBC patients receiving neoadjuvant therapy (2).

The dynamic equilibrium of the tumor immune microenvironment (TIME) critically determines the activation or suppression of anti-tumor immunity (3). Emerging evidence indicates that TIME heterogeneity in TNBC serves as a pivotal determinant of immunotherapy efficacy. This heterogeneity not only affects initial treatment response but also correlates closely with drug resistance and prognosis (4–6). Within this context, Tertiary lymphoid structures (TLS) and tumor-infiltrating lymphocytes (TILs) play vital roles in TNBC immunotherapy (7–11).

TLS refer to ectopic lymphoid organs formed in non-lymphoid tissues under pathological conditions, typically induced at sites of chronic inflammation such as tumors or autoimmune diseases. TLS are classified into three maturation stages: early/immature TLS, primary follicle-like TLS, and secondary follicle-like TLS. Substantial evidence demonstrates that TLS mediate anti-tumor immunity and correlate positively with favorable prognosis in multiple malignancies (including breast, colorectal, and melanoma cancers), serving as independent predictors of both prognosis and immunotherapy response (12–14). Paradoxically, TLS presence is associated with poor prognosis in specific cancer types, potentially attributable to their anatomic distribution within tumor tissues and compositional heterogeneity. TILs constitute immune cells within the tumor microenvironment that influence tumor progression and treatment response. TILs can recognize and eliminate tumor cells through anti-tumor effects; conversely, certain subsets (particularly regulatory T cells) may suppress immune responses and promote immune evasion. Extensive studies confirm that TIL density, subtype composition, and spatial distribution features represent significant independent prognostic factors in various cancers including colorectal, breast, and melanoma. Tumor-infiltrating T cells have been established as robust prognostic indicators across solid tumors (10, 15, 16).

Deep learning advances have dramatically enhanced AI applications in pathological image analysis, particularly in quantitative and qualitative assessment. For instance, AI model demonstrated precise TLS identification in gastrointestinal tumor samples with high concordance to pathologists’ evaluations (17). Another pan-cancer study developed an automated TLS prediction pipeline enabling efficient cross-cancer TLS detection, where TLS percentage within tissues predicted immunotherapy response (18). AI-driven TIL quantification has established correlations between lymphocyte quantity, density, spatial distribution, and therapeutic response/prognosis across multiple cancers (19–22). However, current research predominantly focuses on AI-based quantification of TLS/TIL of entire whole-slide images (WSI) for prognostic analysis, while studies directly investigating the spatial relationships between tumor cells and TILs cells in HE-stained images have been relatively scarce. Furthermore, studies using AI to investigate TNBC response to neoadjuvant therapy focuses mainly on chemotherapy-treated cases, whereas researches using cases treated with chemo-immunotherapy remains relatively limited.

To address these gaps, we developed two automated AI models for TLS identification and TIL classification, enabling accurate detection and quantification of TLS and TILs in HE-stained TNBC samples. Through comprehensive quantitative analysis of the spatial distribution of TILs and the spatial distribution relationships between TILs and tumor cells, we investigated the correlation between these features and chemo-immunotherapy efficacy in TNBC patients, elucidating underlying biological mechanisms.

## 2 Methods

### 2.1 Data preparation

This study utilized two distinct sample cohorts sourced from independent institutions. All HE-stained slides were digitized at 200× magnification using KF-PRO-120 scanners (KONFOONG Biotech, China), with stringent quality control implemented throughout the sectioning-to-imaging workflow to exclude out-of-focus regions, artifacts, and poorly stained sections. A cohort of 383 tumor specimens was obtained from 3D Medicines Inc. (China). This cohort comprised lung cancer (n=104), gastric cancer (n=233), triple-negative breast cancer (TNBC; n=36), and colorectal cancer (n=10) specimens. The TNBC specimens formed an independent testing set, while specimens from the other cancer types constituted the model development dataset. For the development of tumor segmentation and TIL quantification models, 50 TNBC surgical specimens from Zhejiang Cancer Hospital were collected. Concurrently, 32 pretreatment core needle biopsy samples were obtained from patients with histologically confirmed TNBC who subsequently received neoadjuvant chemo-immunotherapy. For each case, the corresponding post-treatment surgical resection specimen was evaluated by experienced pathologists to determine the Miller-Payne (MP) grade, based on the reduction in tumor cellularity compared to the pretreatment biopsy. The digitized pretreatment biopsy slides were used for all subsequent AI-based spatial feature extraction and correlation analysis with the MP response grade. This study was approved by Medical Ethics Committee of Zhejiang Cancer Hospital (Approval No.: IRB-2024-9(IIT)).

### 2.2 TLS recognition framework

TLS identification was implemented via a two-step deep learning workflow: a segmentation model to delineate TLS contours and a classification model to categorize TLS into mature and immature subtypes. To establish the training datasets for both models, experienced pathologists annotated TLS regions on HE slides as ground truth. During annotation, pathologists classified TLS as primary follicle-like or secondary follicle-like based on established H&E morphological criteria. Annotators also considered architectural features suggestive of B-cell follicles and follicular dendritic cell networks to inform the distinction. All annotations underwent cross-validation by a secondary reviewer. Two specialized datasets were curated: 1) 5,357 TLS and 2,770 background patches at 3× magnification (512×512 pixels), along with another 3,963 TLS and 2,565 background patches at 10× magnification (1024×1024 pixels) for TLS segmentation; 2) 2,261 patches of primary follicle-like TLS and 1,702 patches of secondary follicle-like TLS (representing immature and mature states, respectively) for TLS classification.

**Figure 1:**
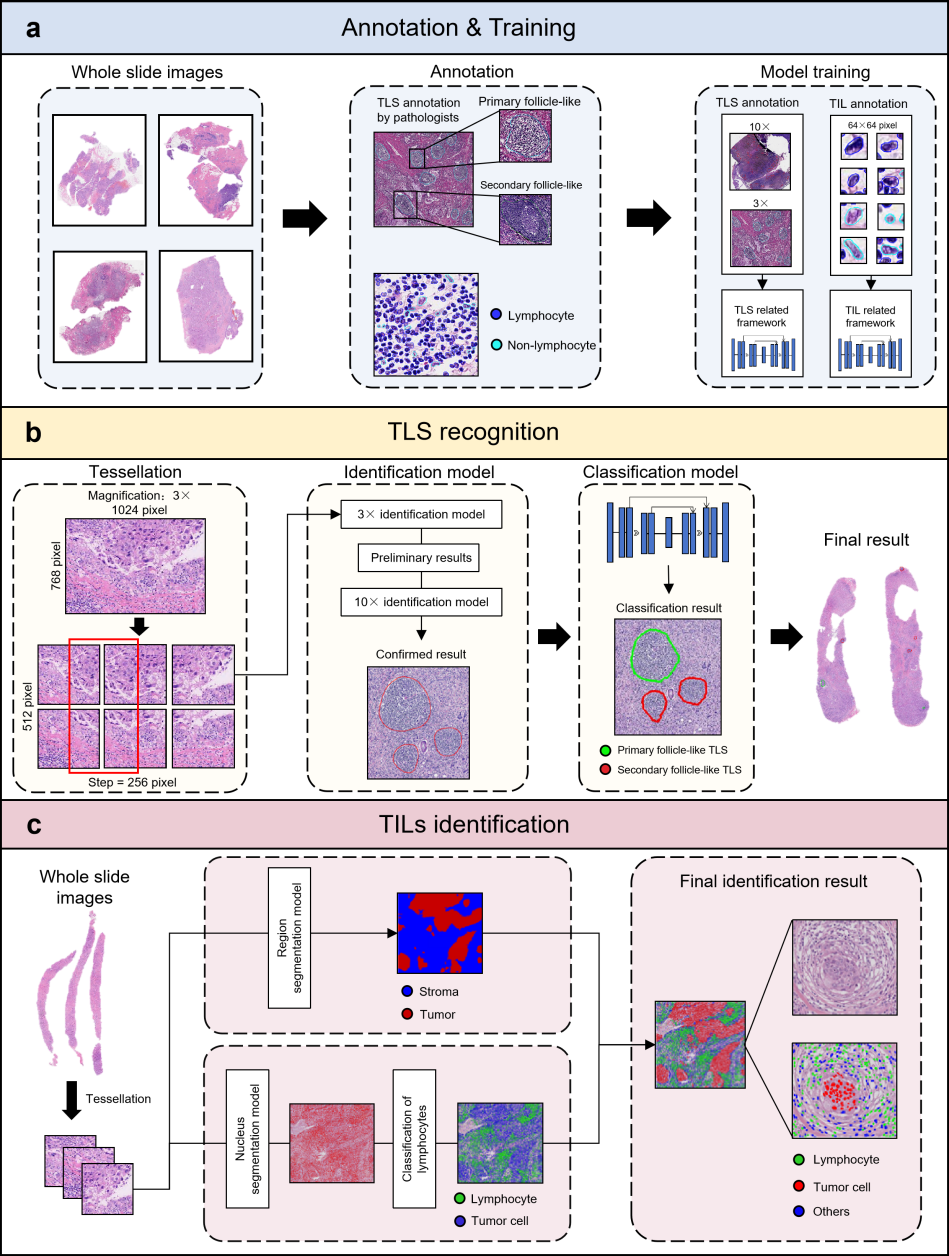
Integrated computational framework for TLS recognition and TILs identification. **(a)** Pathologist-annotated WSIs delineate primary follicle-like (germinal center-negative) and secondary follicle-like (germinal center-positive) TLS structures, alongside lymphocyte/non-lymphocyte identification. Supervised training employs U-Net architecture with MobileNetV2 backbone, with TIL identification model utilizing 64×64-pixel TIL annotation patches and TLS recognition model utilizing variable-sized segmentation patches across 3×/10× magnifications. **(b)** Computational analysis initiates with 3× WSI tessellation (512×512-pixel patches, 256-pixel stride) for preliminary TLS identification. Candidate regions undergo 10× validation (1024×1024 inputs) to exclude false positives, followed by maturity classification outputting spatially mapped primary (green contours) and secondary TLS (red contours). **(c)** Region segmentation partitions tissue into tumor (red) and stromal (blue) compartments. Nucleus segmentation delineates individual cells, while phenotypic classification distinguishes lymphocytes (green), tumor cells (red), and non-lymphoid stromal cells (blue) through morphological and contextual features. (TLS, tertiary lymphoid structure; TILs, tumor-infiltrating lymphocytes; WSI, whole-slide image).

A U-Net architecture with a MobileNetV2 encoder backbone was implemented for TLS segmentation (**Figure 1a**). Two models were trained using the data at different magnifications (3× and 10×) with batch size of 4 and 2 respectively. Both models utilized the Focal Tversky loss function and were evaluated using the Dice coefficient. Training employed an 8.5:1.5 training-validation split. Hyperparameters included a learning rate of 0.0001 and 500 epochs. Data augmentation comprised rotation, flipping, and color jittering.

The TLS maturity classification model was trained with cross-entropy loss using MobileNet V2 architecture. Hyperparameters included a batch size of 4, a learning rate of 0.0005, and 200 epochs. The dataset was split 8:2 (training: validation). Model performance was assessed using F1-score and accuracy. Augmentation incorporated blurring techniques. Final models for both tasks were selected based on optimal validation performance.

The TLS prediction cascade progressed through sequential stages of whole-slide HE image analysis:

WSIs were tiled at 3× magnification with 50% overlap. The 3× segmentation model identified candidate TLS regions within these image patches (**Figure 1b**). Then, coordinates of TLS-positive regions were mapped to corresponding 10× magnification regions. The 10× segmentation model then refined TLS identification at this higher resolution. At last, verified TLS regions underwent classification to determine maturity status (primary or secondary follicle-like structures). This process generated spatially resolved annotations across the entire slide.

### 2.3 Integrated TILs identification framework

TIL quantification was achieved through a multi-model pipeline integrating nuclear segmentation, cell classification, and tumor segmentation models (**Figure 1c**). The tumor segmentation model was trained using an established U-Net framework on APTime analysis software (SODA Data Technology Inc. Shanghai) with 512×512-pixel image patch as input (the training dataset contain 2,138 tumor region-annotated patches with 1024×1024-pixel size).

Both nuclear segmentation and classification models were trained on 256×256-pixel image patches (21,889 annotated lymphocytes and 19,208 non-lymphoid cells) extracted from 20× magnification HE-stained images. Each annotated nucleus was center-cropped into 64×64-pixel image patches prior to classification model training. All the annotations were finished by two board-certified pathologists.

A StarDist model was trained for nuclear segmentation (23). The StarDist model was trained *de novo* on our annotated dataset, without using a pre-trained base model, to ensure optimal performance on the specific morphology of our HE-stained images. For cell classification model development, the MobileNet V2 architecture - consistent with the TLS framework - was employed. These models were optimized with a learning rate of 0.0005 and a batch size of 8, aiming to minimize the categorical cross-entropy loss. The dataset was partitioned randomly, allocating 70% for training and 30% for validation. During the 200-epoch optimization process, data augmentation was applied via random rotation and flipping. The final model was selected based on maximal validation accuracy.

The TILs identification workflow comprises four sequential computational stages: (1) tumor segmentation to partition tissue into tumor and stroma compartments; (2) nuclear segmentation to identify all nuclear structures; (3) lymphocyte classification using the classification model; and (4) phenotype categorization, where each cell is classified as lymphocyte, tumor cell, or other cell by integrating outputs from the tumor segmentation and lymphocyte classification models. For all downstream analyses, lymphocytes were quantified irrespective of their location, including those within TLS and larger lymphoid aggregates. This approach was chosen to capture the full spatial organization of lymphocytes in the tumor microenvironment.

### 2.4 Spatial analysis

Proximity analysis quantified target cell (TC) density within concentric circular zones (radius range: 10, 20, 30, 50, 100, and 200 μm) centered on reference cells. Evaluations were conducted separately with either tumor cells or lymphocytes as reference cells, assessing lymphocyte distribution around tumor cells (refer as lymphocyte-tumor configuration) and tumor cell distribution around lymphocytes (refer as tumor-lymphocyte configuration). Aggregate analysis defined cellular aggregates as clusters of phenotypically identical cells with pairwise intercellular distances <20 μm. Aggregate size thresholds ranged from n = 2 to 5 cells. Other analysis measured counts and densities of both tumor cells and lymphocytes within tumor region and stromal region.

All derived metrics underwent correlative, differential, and feature importance analysis against the MP histopathological grading system (a validated five-tier framework quantifying pathological response to neoadjuvant immune checkpoint therapy in breast cancer). MP grade 1 exhibited no significant reduction in malignant cells; MP grade 2 demonstrated ≤30% neoplastic cellular reduction; MP grade 3 showed 30-90% reduction; MP grade 4 exceeded 90% reduction; and MP grade 5 achieved pathological near-complete response with no residual invasive carcinoma in tumor beds (allowing for *in situ* carcinoma presence).

### 2.5 Random forest modeling

Random forest classifiers were implemented for both feature importance analysis and therapeutic response prediction using the randomForest package (v4.7-1.1). Node size was optimized at 5 to balance model complexity and generalizability, while feature randomization at each split employed √p variables (where p = total predictors). For MP grade prediction, a separate forest of 500 trees was constructed using hyperparameters, incorporating proximity features, aggregation features, and other cell quantification features. Model performance was assessed via stratified 5-fold cross-validation repeated 3 times, with out-of-bag error estimation serving as an internal validation metric. Variable importance was quantified through mean decrease in Gini impurity, with higher values indicating greater discriminatory power for MP stratification.

### 2.6 Statistical analysis

All statistical analyses were performed using Python version 3.13.5. P-values from correlation analyses and group comparisons were adjusted using the Benjamini–Hochberg False Discovery Rate (FDR) procedure. Statistical significance was defined as FDR-adjusted p<0.05 (two-sided). Model performance of TLS segmentation and lymphocyte classification was quantified utilized accuracy. Wilcoxon rank-sum testing (Mann-Whitney U test) was used for non-normally distributed continuous variables. Spearman’s correlation (R) assessed monotonic relationships between continuous variables, where R = 1 indicates perfect positive monotonicity; R=-1 indicates perfect negative monotonicity; and R = 0 indicates no monotonic association. Feature importance analysis was conducted using random forest classifiers, which aggregated predictions from multiple decision trees while evaluating feature contributions through Gini impurity reduction.

## 3 Results

### 3.1 Study cohorts

The study investigating the association between histopathological features and treatment response included 32 patients with histologically confirmed TNBC who received neoadjuvant chemotherapy combined with immunotherapy (**Supplementary Table S1**). The cohort had a median age of 47 years (range: 24–70). All patients were treated using standard taxane and/or anthracycline-based chemotherapy regimens integrated with PD-1 inhibitors. Pathological responses were assessed on post-treatment surgical specimens using the MP grading system by comparing them to the corresponding pretreatment biopsies. The distribution demonstrated significant responses (Grade 4-5) in 34.4% (n=11) of patients. Detailed clinicopathological characteristics of the cohort are provided in **Supplementary Table S1**. A CONSORT-style patient workflow diagram illustrating sample inclusion and exclusion for model development and response correlation analysis is provided in **Supplementary Figure S1**.

### 3.2 Performance validation of TLS and TILs identification models

**Figure 2:**
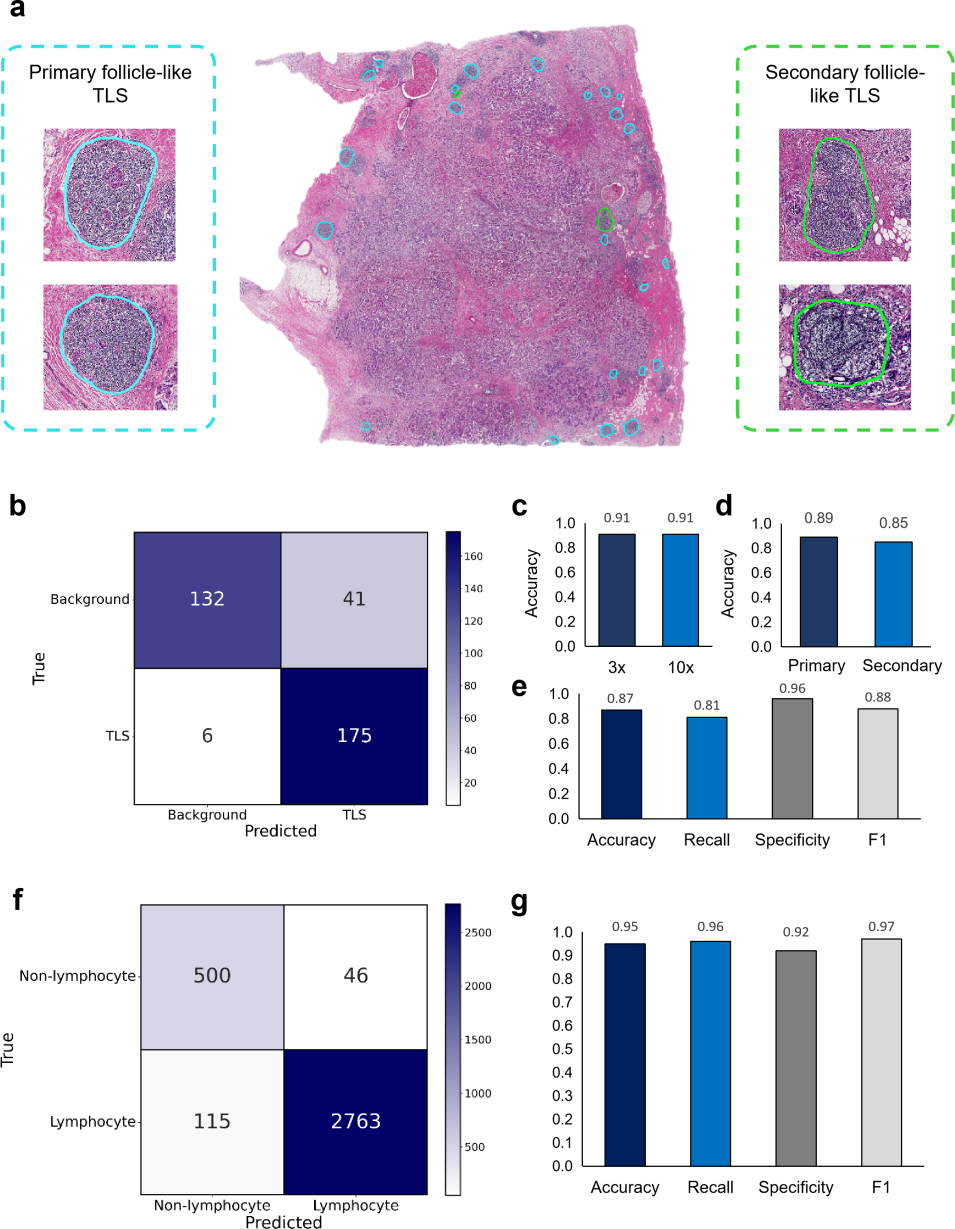
Performance validation of AI-driven TLS recognition framework. **(a)** HE-stained tissue section demonstrating primary follicle-like TLS (blue contours) lacking germinal centers and secondary follicle-like TLS (green contours) with organized follicular architecture. **(b)** Confusion matrix comparing AI-predicted TLS against pathologist-verified ground truth. **(c)** Quantitative performance evaluation across optical resolutions with 3× and 10× objectives. **(d)** Quantitative performance evaluation for primary versus secondary TLS subtypes. **(e)** Accuracy, recall, specificity, and F1-score confirming robust discriminatory power of the TLS identification framework in independent dataset of 36 breast cancer samples. **(f)** Confusion matrix comparing AI-predicted lymphocyte classifications against pathologist-verified ground truth. **(g)** Accuracy, recall, specificity, and F1-score confirming robust discriminatory power of the TIL classification framework.

The TLS identification workflow demonstrated robust performance across validation datasets. The initial localization model at 3× magnification achieved 0.91 accuracy for TLS detection, while the subsequent 10× magnification verification model attained equivalent accuracy (0.91) in discriminating bona fide TLS structures (**Figures 2a–c**). Maturity classification of confirmed TLS regions yielded accuracies of 0.89 for primary follicle-like TLS and 0.85 for secondary follicle-like TLS. When applied to the independent dataset of 36 breast cancer samples, model predictions showed substantial concordance with pathologist interpretations (κ= 0.73 ± 0.04), with 0.87 accuracy (95% CI: 0.83-0.90), 0.81 recall (95% CI: 0.75-0.86), 0.96 specificity (95% CI: 0.91-0.98), and 0.88 F1-score (95% CI: 0.86-0.92) (**Figure 2e**). **Supplementary Figure S2** showed some examples that were easily misidentified as TLS.

The optimized TIL quantification framework demonstrated robust classification performance (**Figures 2f, g**) with an accuracy of 0.95 (95% CI: 0.95-0.96) and F1-score of 0.97 (95% CI: 0.97-0.98), while achieving exceptional precision (0.98, 95% CI: 0.98-0.99) and recall (0.96, 95% CI: 0.95-0.97). The model maintained strong specificity (0.92, 95% CI: 0.89-0.94) and substantial inter-rater agreement (κ = 0.83 ± 0.01). Critically, the precise tumor boundary delineation eliminated cellular misclassification in tumor regions, preventing misclassification of tumor cells with small size as lymphocytes (**Figure 2**).

Collectively, the multi-resolution TLS recognition framework achieved pathologist-aligned accuracy (>0.85 across subtypes), while the TIL pipeline demonstrates near-perfect cellular phenotyping specificity (0.98). These frameworks provided a validated computational pathology foundation for precision immuno-oncology applications.

### 3.3 Correlation analysis between spatial features and MP

**Figure 3:**
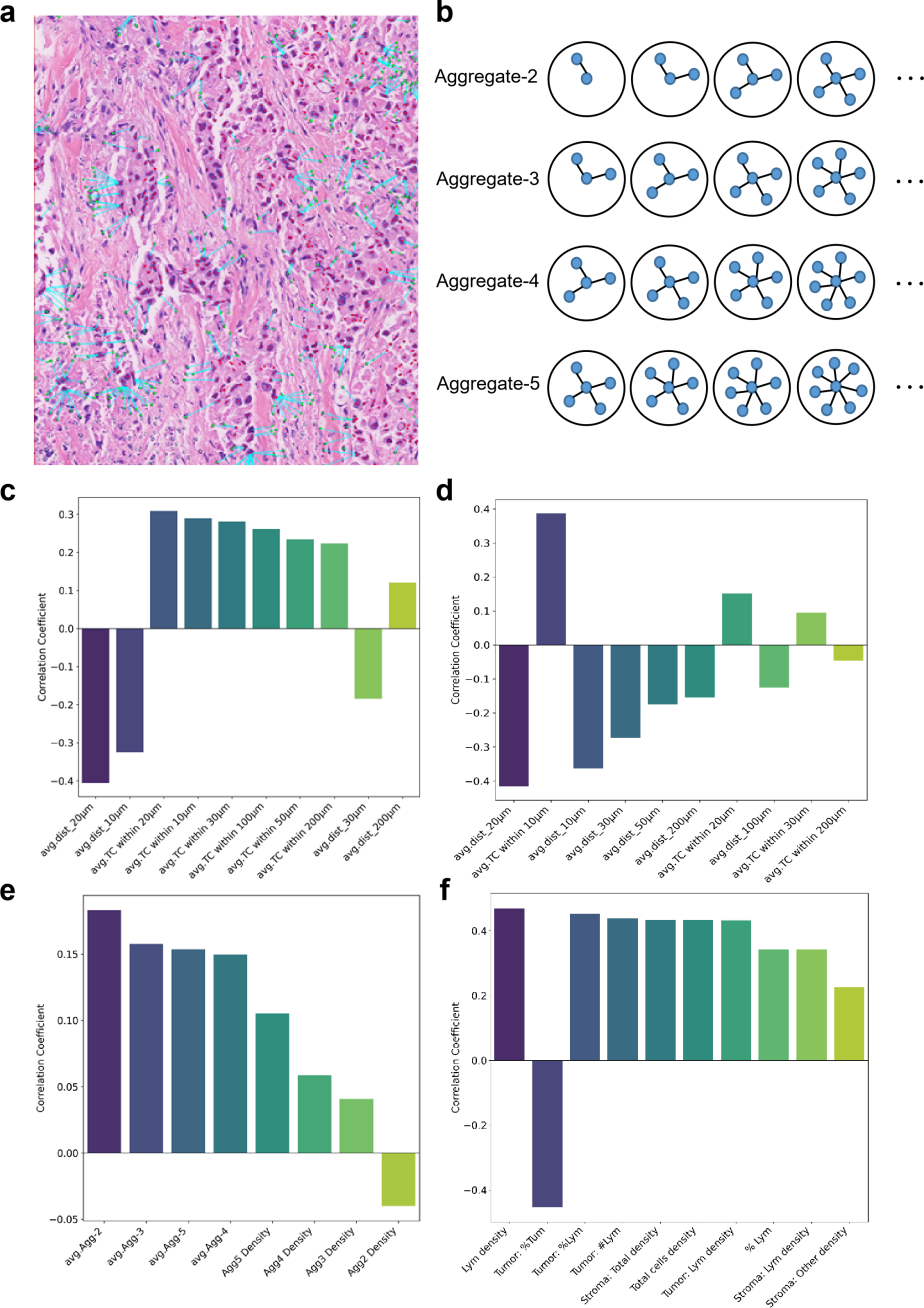
Spatial lymphocyte aggregation patterns and correlative significance with therapeutic response. **(a)** Representation of lymphocyte distribution around tumor cells (cyan arrows). **(b)** Quantitative definition of lymphocyte aggregates. Schematic representation classifying cellular clusters as Aggregate-n (n = 2–5 lymphocytes) based on cellular enumeration and spatial contiguity requirements (pairwise intercellular distance <20 μm). Blue nodes denote individual lymphocytes connected by adjacency vectors (black lines), with circular contours demarcating cluster boundaries. **(c)** Spearman correlation coefficients (ρ) between MP grades and spatial metrics measuring average lymphocyte-tumor distances at progressive radii (10-200 μm). **(d)** Spearman correlation of MP grades with tumor cell density measures within concentric lymphocyte-centered zones (10-200 μm). **(e)** Spearman correlation of MP response with Aggregate-n cellular counts (n = 2-5) and cluster densities. **(f)** Spearman correlations of MP response with cell quantification metrics. (TC, target cells; Lym, lymphocytes; Tumor, tumor cells; TIL, tumor-infiltrating lymphocytes).

Following lymphocyte and tumor cell quantification using the TIL framework, we computed three categories of spatial distribution features: (1) spatial proximity metrics with lymphocytes or tumor cells as reference points; (2) lymphocyte aggregation characteristics; and (3) cell quantification parameters. Comprehensive feature definitions are cataloged in **Supplementary Table S2**. Within aggregation metrics, aggregate_2 denotes clusters containing ≥2 lymphocytes with pairwise intercellular distances <20 μm, with higher-order aggregates (aggregate_n) following analogous criteria (**Figures 3a, b**). Bidirectional proximity analysis revealed significant correlations between lymphocyte-tumor spatial distributions and MP grades. Lymphocyte-centric (meaning lymphocytes are reference cells) assessment demonstrated that shorter lymphocyte-tumor distances within critical radii (Average Distance_10: R = -0.36, p = 0.0410; Average Distance_20: R = -0.42, p = 0.0178) strongly associated with higher MP grades (**Figure 3c**). Notably, Lymphocyte enrichment within 200 μm, 100 μm, 50 μm, 30 μm, 20 μm, and 10 μm radii of tumor cells exhibited a moderate positive correlation with MP grade elevation (**Figure 3c**).

Tumor-lymphocyte analysis (**Figure 3d**) revealed similar negative correlations for tumor cell proximity and MP grade (Average Distance_10: R = -0.32, p = 0.0286; Average Distance_20: R = -0.40, p = 0.0178). And tumor cell enrichment within 10 μm range of lymphocyte cells also exhibited moderate positive correlation with MP grade elevation (**Figure 3c**). However, aggregation metrics demonstrated that lymphocyte aggregate density didn’t significantly stratified MP response (**Figure 3e**), with Aggregates-2 (R = 0.18, p = 0.3158) and Aggregates-3 (R = 0.16, p = 0.3889). Consistent with these findings, stromal lymphocyte density exhibited a moderate correlation with therapeutic response (R = 0.47, p = 0.0044), further supporting the functional significance of lymphocyte spatial organization in anti-tumor immunity (**Figure 3f**).

### 3.4 Analysis of the importance of spatial distribution features

**Figure 4:**
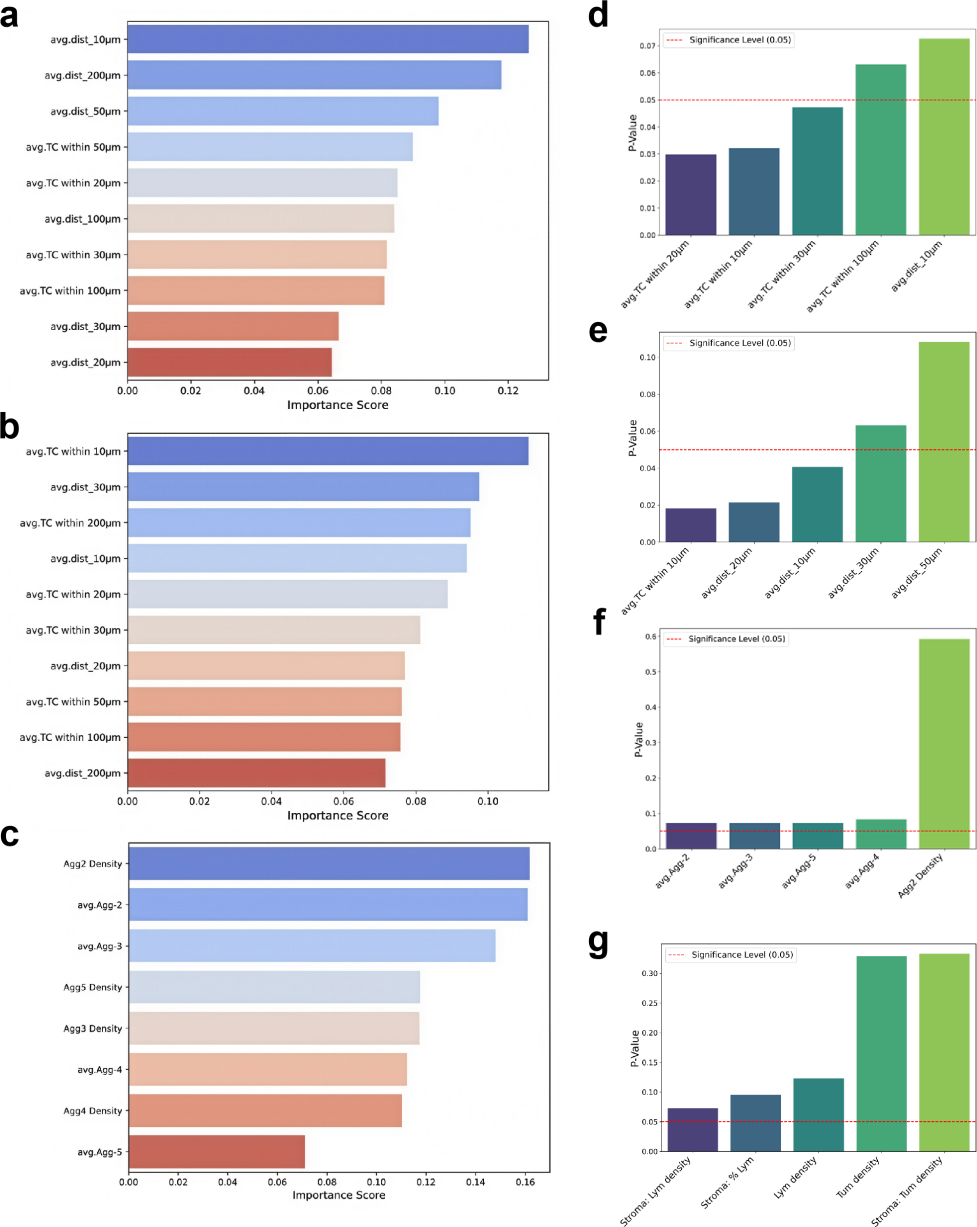
Predictive feature analysis and inter-group differentiation of tumor immune microenvironment spatial metrics. Feature importance for therapeutic response prediction: Random forest-derived importance scores quantifying the contribution of microenvironmental metrics to MP grading. **(a)** Lymphocyte-centric proximity features **(b)** Tumor-centric proximity features **(c)** Lymphocyte aggregation features. **(d–g)** Inter-group differential significance: Mann–Whitney U test p-values assessing statistical disparity between MP1–3 and MP4–5 cohorts across feature classes: **(d)** Lymphocyte-centric proximity, **(e)** Tumor-centric proximity, **(f)** Aggregation parameters, and **(g)** cell quantification metrics. The dashed red line (p=0.05) denotes significance threshold. Lower p-values indicate stronger statistical discrimination.

Random forest-based feature importance analysis quantified the discriminatory power of spatial metrics for MP grading. In lymphocyte-centric evaluation, the average lymphocyte-tumor distance within 10 μm demonstrated the highest predictive influence (Importance score=0.14) (**Figure 4a**), indicating that MP stratification is critically dependent on lymphocyte-tumor spatial proximity. Tumor-centric analysis conversely identified average tumor cell count within 10 μm as the predominant feature (Importance score =0.11) (**Figure 4b**), suggesting that MP progression correlates with the aggregation of tumor cells. Aggregation metrics further established low lymphocyte aggregates as superior discriminators (**Figure 4c**), with Aggregates-2 counts (Importance score =0.16) and density (Importance score =0.16) exceeding contributions from higher aggregates (Aggregates-3 count/density score =0.14/0.12; Aggregates-4/5 score <0.12). These results collectively identify lymphocyte-tumor spatial adjacency and small-cluster coordination as key determinants of therapeutic response.

### 3.5 Comparative differential analysis between MP groups

To address disparities in sample sizes and heterogeneity in pathological response across individual MP grades, patients were stratified into comparative cohorts: MP1-3 (low/intermediate response) versus MP4-5 (high response), enabling statistical evaluation of quantitative metrics between these groups. Comparative analysis (MP1–3 vs. MP4-5) revealed significant spatial stratification across spatial distribution metrics. Lymphocyte-centric proximity assessment demonstrated substantial inter-group differences in lymphocyte-tumor distances within critical radii (10/20/30µm: p<0.05) (**Figure 4d**). Tumor-centric analysis confirmed similar distance differentials (10/20/30 µm: all p<0. 1) alongside elevated tumor cell density within 10 µm in MP4-5 (p=0.018) (**Figure 4e**). Aggregate metrics showed progressive elevation in cluster cellularity across orders (Aggregates-2/3/4/5: p<0.1) (**Figure 4f**). Stromal compartment analysis further identified increased lymphocyte density (p<0.1) and lymphocyte count (p<0.1) in high grade MP cohorts (**Figure 4g**), establishing spatial distribution features as robust discriminators of therapeutic response.

### 3.6 Predictive modeling of MP grading using tumor microenvironment features

**Figure 5:**
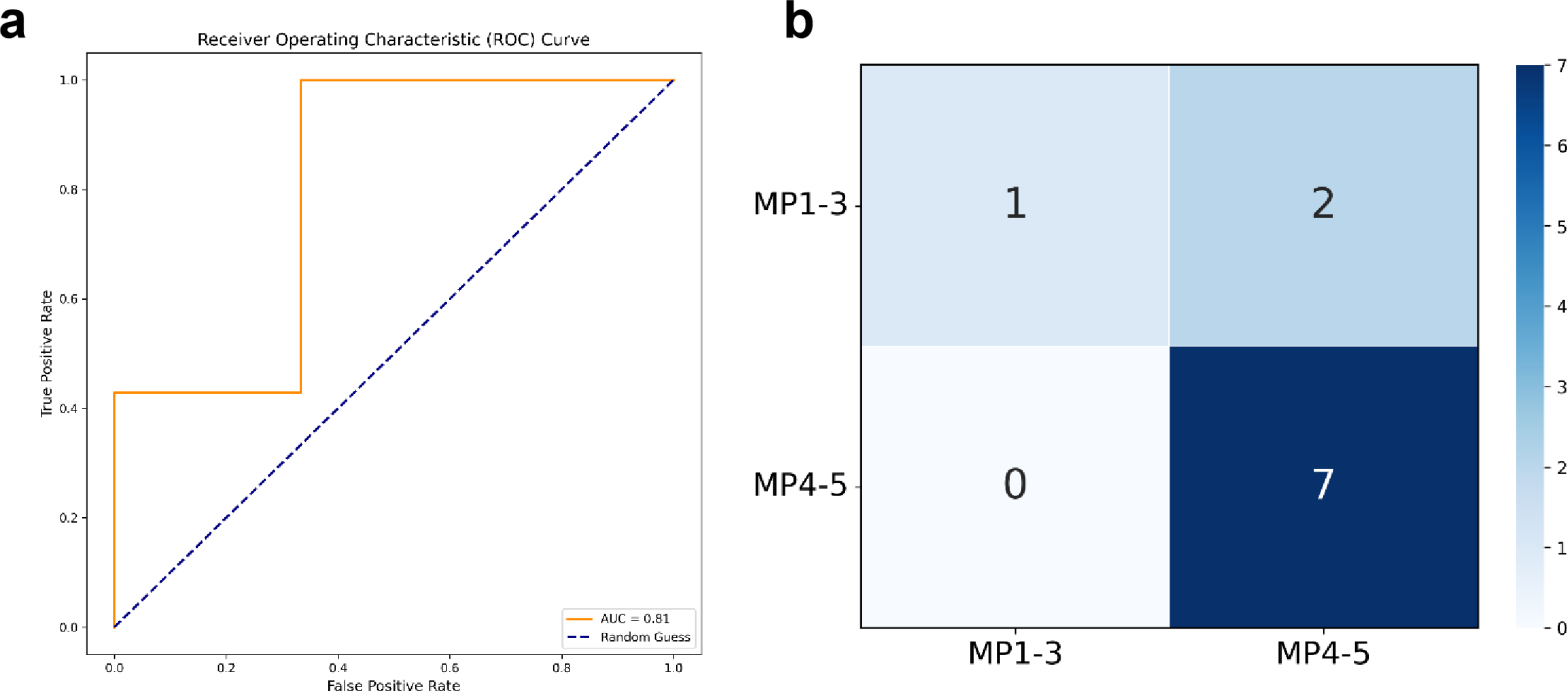
Performance evaluation of feature-optimized random forest classifier for MP response prediction. **(a)** Receiver operating characteristic (ROC) curve from repeated 5-fold cross-validation (best across 15 test folds), for distinguishing high therapeutic responders (MP4-5) from low/intermediate responders (MP1-3). **(b)** Confusion matrix from the best-performing fold of the cross-validation, illustrating the model’s predictive performance under optimal conditions (X-axis, predicted; Y-axis, true).

To investigate the predictive capacity of spatial distribution features for MP grading in TNBC after neoadjuvant chemo-immunotherapy, we developed a random forest classifier incorporating multidimensional parameters including lymphocyte-tumor spatial proximity metrics, aggregation characteristics, and cell quantification profiles. This integrated model successfully achieved quantitative MP grade prediction. The classifier demonstrated robust performance on the test cohort (AUC = 0.81; 95% CI: 0.76-0.86), substantially outperforming random classification baselines (DeLong test, p = 2.3 × 10^-5^) as evidenced by ROC analysis (**Figures 5a, b**). These results validate the effectiveness of multidimensional spatial feature integration, confirming that combining parameters across 10 - 200 μm scales overcomes limitations inherent in single-scale analysis while optimizing model performance balance and generalizability.

## 4 Discussion

The aggressive clinical behavior of TNBC and its limited response to conventional therapies underscore the therapeutic significance of immune checkpoint inhibitors, which have demonstrated superior efficacy in neoadjuvant settings. This aligns with growing evidence establishing the tumor immune microenvironment as a critical determinant of treatment outcomes. Consistent with prior findings showing that CD8+ T-cell proximity to tumor cells predicts immunotherapy response (24), our spatially-resolved analysis of preoperative biopsies revealed three insights: first, stromal lymphocyte density and percentage exhibited positive correlation with MP pathologic response grades; second, diminished lymphocyte-tumor distances within critical radii (10–30μm) strongly associated with higher MP grades regardless of reference object, This implies that the closer proximity between the two cell types correlates with stronger interactions and a more potent anti-tumor immune response. These findings collectively support current paradigms by demonstrating that beyond quantitative metrics, the spatial topography of immune-tumor interactions, particularly the distance-dependent immune effector functions, constitutes a new dimension of immunobiological regulation.

Our findings also align with and extend recent high-dimensional spatial profiling studies which demonstrated that the proximity of proliferative CD8+ TCF1+ T cells to tumor cells was a key spatial predictor of immunotherapy response in TNBC (25). While our H&E-based approach cannot resolve such specific phenotypes, our observation that shorter lymphocyte-tumor distances (within 10–30μm) strongly correlates with response is consistent with their findings, suggesting that our AI model is capturing a surrogate of this crucial biological interaction.

Our deep learning framework addressed two key microenvironmental components: TILs quantification confirmed established density-immunotherapy response relationships, while the novel TLS detection model demonstrated cross-cancer applicability despite the scarcity of TLS structures in 32 biopsy samples. The latter observation likely reflects sampling limitations rather than biological absence, suggesting surgical samples may better elucidate TLS clinical relevance as indicated by prior prognostic studies (7, 8, 26). Notably, through the TLS identification model, we identified multiple TLS structures in an MP5-grade sample, including mature TLS (**Supplementary Figure S3**), which is believed to possess stronger anti-tumor activity and prognostic predictive value. In this sample, the infiltration of lymphocytes is also relatively abundant, suggesting that scattered lymphocytes and highly differentiated TLS structures may collaborate in exerting anti-tumor activity. Notably, lymphocyte aggregation metrics functionally approximated TLS biology, with cluster-cellarity differentiations implicating coordinated immune cell cooperation in anti-tumor immunity. The operational integration of these spatial and quantitative signatures within a random forest classifier achieved significant predictive discrimination (AUC = 0.81; 95% CI: 0.76–0.86), highlighting the translational potential of multiplexed microenvironmental profiling.

Several limitations warrant consideration. First, this is an exploratory study with a biopsy-based cohort of 32 patients, which inherently constrained spatial heterogeneity assessment; external validation in a larger, multi-institutional cohort is required to confirm the generalizability of our findings. Second, our analysis is limited by the use of H&E staining, which precludes phenotypic differentiation of lymphocyte subpopulations. For instance, a high density of lymphocytes in close proximity to tumor cells could, in theory, represent a suppressive population (e.g., FoxP3+ regulatory T cells). However, the strong positive correlation of this spatial feature with favorable response (MP4-5) in our study suggests that the lymphocytes we are detecting are predominantly effectors. Third, while our pipeline integrates tumor-stroma segmentation, we did not perform a formal compartment-specific comparison of intra-tumoral versus stromal lymphocytes, as the model cannot reliably differentiate lymphocytes truly infiltrating tumor nests from those adjacent to tumor cells in densely packed regions. Nevertheless, our spatial proximity metrics within 10–30 μm indirectly capture the degree of lymphocyte infiltration into tumor regions, with shorter distances reflecting closer physical association with tumor cells. Fourth, while our TLS maturity classification relied on H&E morphology validated by expert pathologists, immunophenotyping with lineage-specific markers would provide a more definitive and functionally informative characterization of TLS maturation. Future studies incorporating surgical samples with multiplex immunohistochemistry could delineate spatial-immune cell interactions with greater resolution, while integrating clinicopathologic variables (e.g., molecular subtypes) may enhance model precision. Nevertheless, this AI-driven analytical pipeline establishes an actionable methodology for quantifying multidimensional microenvironmental determinants, demonstrating that spatial biomarkers, particularly immune-tumor proximity, may refine response prediction and inform therapeutic strategies. As HE-stained samples are routine and readily available in clinical pathology, the computational framework thus represents a significant advancement toward precision immuno-oncology applications in clinical diagnostics and drug development.

## Data Availability

The original contributions presented in the study are included in the article/**Supplementary Material**. Further inquiries can be directed to the corresponding authors.
