# Supplementary material for: AI-driven spatial analysis of tumor-infiltrating lymphocytes predicts chemo-immunotherapy response in triple-negative breast cancer
Source: Front Immunol. 2026 Apr 16;17:1797753. doi: 10.3389/fimmu.2026.1797753 (PMC13128803; doi:10.3389/fimmu.2026.1797753)
Supplement: Supplementary file 3 [file Supplementaryfile1.docx]

Supplementary Material


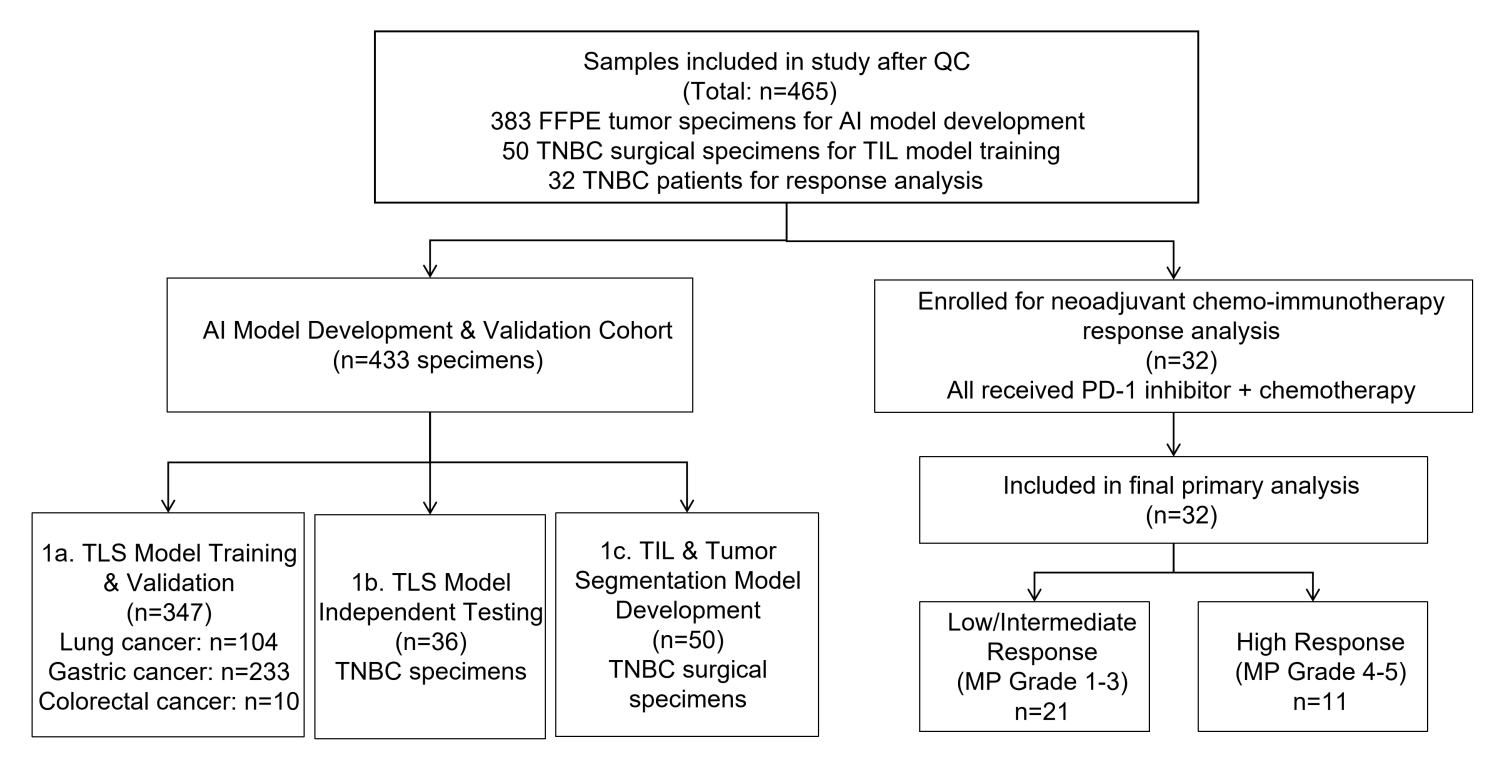


**Supplementary Figure S1.** CONSORT flow diagram of study enrollment and cohort allocation.

This diagram illustrates the eligibility assessment, screening, and cohort distribution of 465 specimens/participants enrolled in this study. All 465 cases passed quality control (QC) and eligibility screening with no exclusions, and were allocated to two core sets: the AI model development & validation cohort (n=433, for TLS and TIL model training, validation and independent testing), and the neoadjuvant chemo-immunotherapy response analysis cohort (n=32 TNBC patients treated with PD-1 inhibitor plus chemotherapy, all included in the final primary analysis). Pathological response stratification by MP grade was performed in the TNBC independent testing cohort.


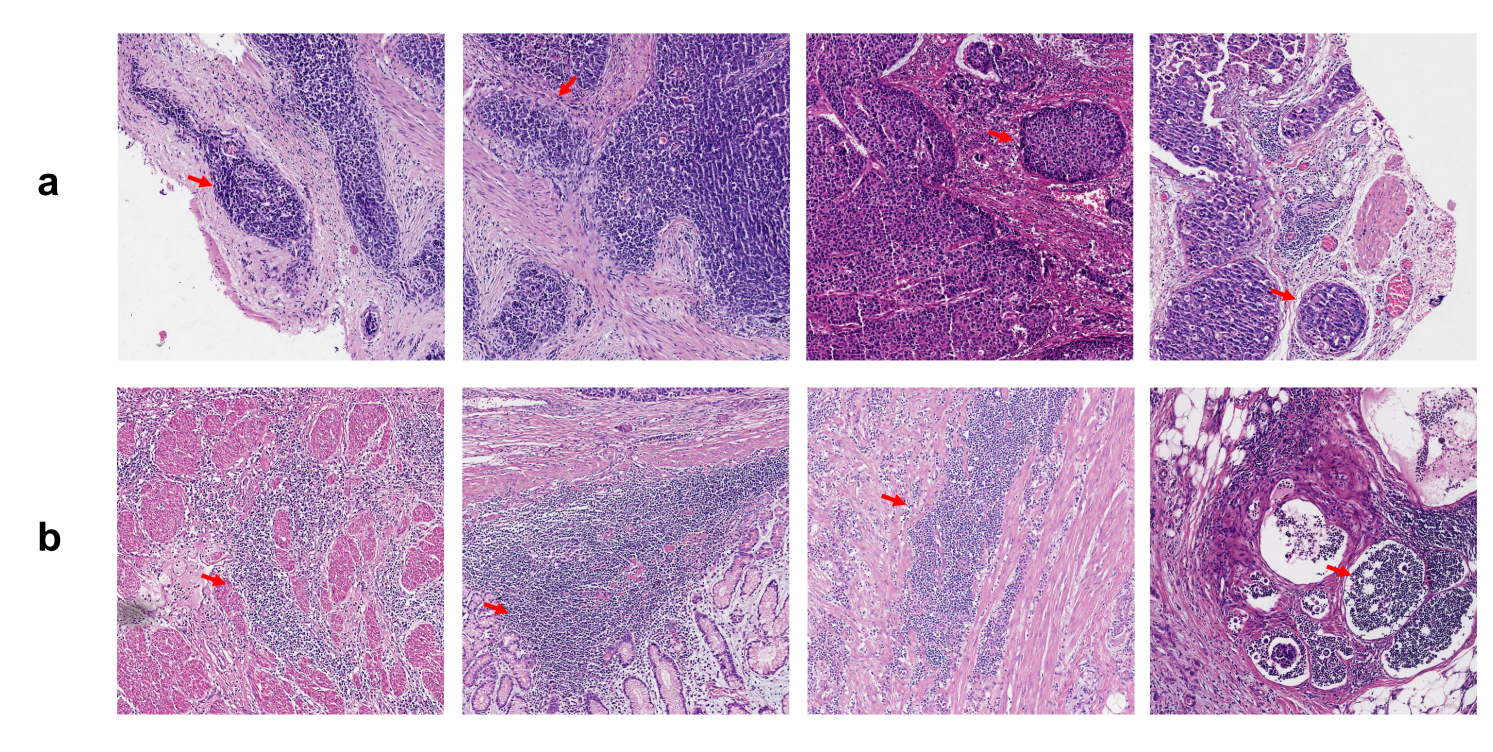


**Supplementary Figure S2.** Examples of false-positive regions prone to misidentification as TLS. (a) Tumor cell regions with small cell volume and TLS-like contour, which are common false-positive targets for TLS identification. (b) Dense lymphocyte aggregation regions without the typical structure of TLS, which are easily misrecognized as genuine TLS. All images are hematoxylin-eosin (HE) stained sections.


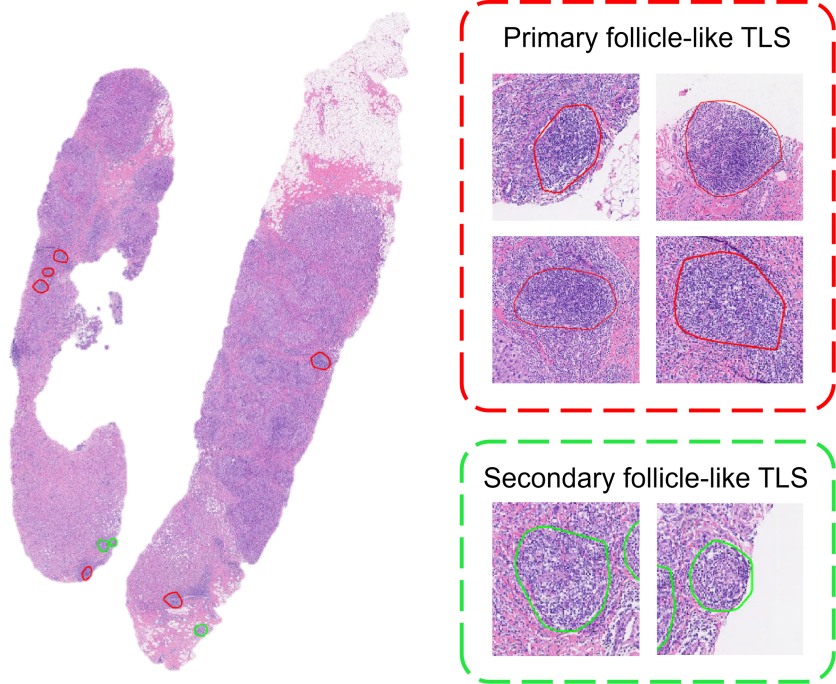


**Supplementary Figure S3.** Supplementary Case Report: Spatial Profiling of TLS in an MP5 Treatment Responder.

Quantitative analysis of a representative core biopsy sample from a TNBC patient achieving pathological complete response (MP grade 5) revealed elevated TLS density within the tumor microenvironment. HE sections demonstrated dense lymphocytic infiltration featuring 9 identifiable TLS structures distributed throughout the tumor bed. Pathologist review confirmed distinct compartmentalization with 6 primary follicle-like TLS structures, alongside 3 secondary follicle-like TLS structures. Limited quantitative analysis indicated higher TLS frequency relative to typical high-response specimens, though formal density metrics were precluded by sample constraints.
